# Covariate and multinomial: Accounting for distance in movement in capture–recapture analyses

**DOI:** 10.1002/ece3.4827

**Published:** 2019-02-05

**Authors:** Loreleï Guéry, Lauriane Rouan, Sébastien Descamps, Joël Bêty, Albert Fernández‐Chacón, Grant Gilchrist, Roger Pradel

**Affiliations:** ^1^ Département de Biologie Chimie et Géographie Université du Québec à Rimouski, Rimouski, Canada – Centre d'Etudes Nordiques Laval Québec Canada; ^2^ CIRAD – Biological Systems Department UMR108 Amélioration Génétique et Adaptation des Plantes méditerranéennes et tropicales Montpellier France; ^3^ Norwegian Polar Institute, Fram Center Tromsø Norway; ^4^ Research Unit of Biodiversity (UO, CSIC, PA) University of Oviedo Mieres Spain; ^5^ National Wildlife Research Centre, Environment Canada Ottawa Canada; ^6^ CEFE UMR 5175 CNRS ‐ Université de Montpellier ‐ Université Paul‐Valéry Montpellier ‐EPHE Montpellier France

**Keywords:** covariate, dependent estimates, link function, multinomial logit, transformations, variance–covariance matrix

## Abstract

Many biological quantities cannot be measured directly but rather need to be estimated from models. Estimates from models are statistical objects with variance and, when derived simultaneously, covariance. It is well known that their variance–covariance (VC) matrix must be considered in subsequent analyses. Although it is always preferable to carry out the proposed analyses on the raw data themselves, a two‐step approach cannot always be avoided. This situation arises when the parameters of a multinomial must be regressed against a covariate. The Delta method is an appropriate and frequently recommended way of deriving variance approximations of transformed and correlated variables. Implementing the Delta method is not trivial, and there is a lack of a detailed information on the procedure in the literature for complex situations such as those involved in constraining the parameters of a multinomial distribution. This paper proposes a how‐to guide for calculating the correct VC matrices of dependant estimates involved in multinomial distributions and how to use them for testing the effects of covariates in post hoc analyses when the integration of these analyses directly into a model is not possible. For illustrative purpose, we focus on variables calculated in capture–recapture models, but the same procedure can be applied to all analyses dealing with correlated estimates with multinomial distribution and their variances and covariances.

## 1 INTRODUCTION

Biologists and ecologists routinely study quantities that are not directly measurable (probabilities of survival, capture, etc.…), especially in wild populations, and thus need to use models to calculate estimates of these quantities. In addition, ecologists are often interested in assessing the effect of covariates on these quantities, such as the comparison of survival before and after the implementation of conservation and management measures. Typically these model‐produced estimates of say survival probability are calculated from the same model and hence not independent, that is, coming with associated variances and covariances. Thus, using the model‐produced estimates in traditional statistical tools, such as for example *t* test (Gossett, 1908), ANOVA (Anderson & Ager, 1978), correlation coefficient, or linear models, highly violates the rule of independence of these tools. The latter therefore becomes inefficient to deal with these dependent estimates because the variance–covariance (VC) structure is ignored.

**Figure 1:**
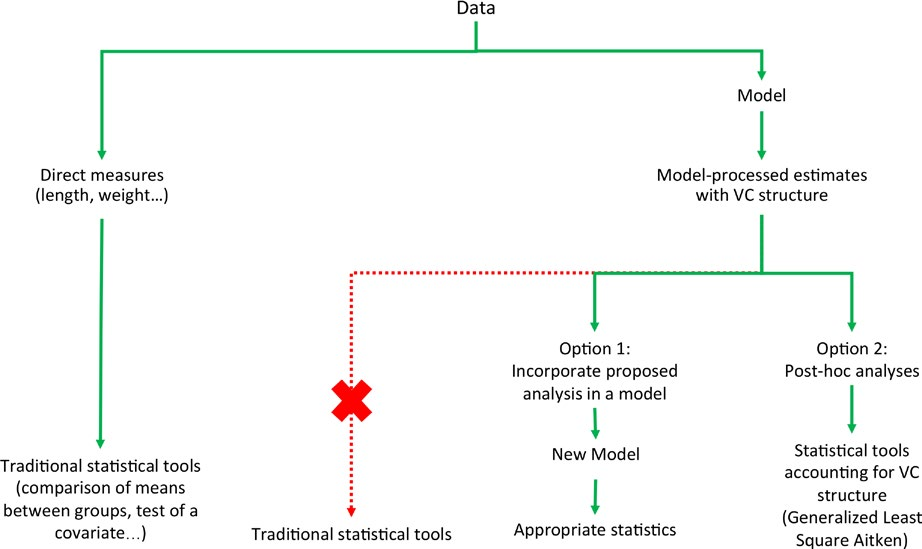
Schematic of statistical approach

The ideal option is to build the proposed analysis in the model (option 1, Figure 1). However, it can sometimes become complicated or even not possible to test hypotheses of interest using the currently available models. Biologists are therefore required to run alternative post hoc analyses on the model‐produced estimates. Some papers previously discussed this issue of doing statistics on statistics in post hoc analysis either in a frequentist framework (Burnham & White 2002; Grosbois et al., 2008; see Dugger et al., 2015 for an example of the use of these models) or in the Bayesian framework (Brooks & Deroba, 2015; Link 1999; Link & Barker 2004) in which a hierarchical approach was recommended (Cooch, Conn, Ellner, Dobson, & Pollock, 2012; Royle & Dorazio 2008; Sutherland, Brambilla, Pedrini, & Tenan, 2016).

A typical example is the study of movement as a function of the distance. In ecology, dispersal of plant seeds or movement of animals are generally more frequent between neighboring sites. To actually test this idea, one may want to relate the frequency of movements between any two locations to the distance separating them. For animals, frequency of exchanges can be measured from capture–recapture (CR) data gathered on a small number of sites. An appropriate multisite model (Cormack, 1964; Jolly, 1965; Nichols & Kendall, 1995; Pradel, Gimenez, & Lebreton, 2005; Seber, 1965), on which we will focus here for illustrative purpose, produces estimates of the probability that an animal moves or stays from a given site of departure toward a site of arrival. The natural idea is then to regress the probability of movement against the distance between the departure and arrival sites directly in the model (option 1, Figure 1). However, because only the surveyed sites are observable as arrival sites, the multisite models can only estimate conditional probabilities of movement (conditional on the fact that the animals do not move out of the set of surveyed sites). As the different possible transition probabilities (including the probability of not departing) must sum to 1, one of those probabilities chosen arbitrarily, but usually the probability of not departing, is the complement of the others. In this case, it is not an actual parameter of the model and cannot be forced into the relationship with the distance. The same problem will arise every time that the quantities to be constrained are parameters with a multinomial distribution, that is, variables with more than two modalities such as the breeding status based on the number of offspring produced or the settlement probabilities in different areas.

To circumvent this difficulty, we propose a second option (option 2, Figure 1), that is, applying a generalized least square (GLS) approach to the estimates produced by the unconstrained multisite model. A proper estimation of the VC matrix is therefore needed as a key element for accounting for statistical uncertainty or correlation in parameters estimates that are included in these post hoc analyses. Some scientific articles commonly apply the Delta method (Dorfman, 1938; Ver Hoef, 2012), a method for deriving variance approximations of transformed and correlated variables. The Delta method theory was fully explained and its use recommended to biologists (Cooch & White, 2016; MacKenzie, 2006; Williams, Nichols, & Conroy, 2002). Powell (2007) described the Delta method in a more accessible way to ecologists, but it was less detailed in describing correlated estimates and providing applications with covariates. The use of the Delta method is rarely made the focal point in scientific papers, much less with multinomial variables. This lack of detailed procedures in the literature could lead to incorrect use where dependent estimates are considered as independent ones (Pape Møller & Szép, 2002). Indeed, Fernández‐Chacón et al. (2013) was, to our knowledge, the only study that used and described a correct use of the variance–covariance (VC) matrix in post hoc analyses to study impacts of a covariate on a parameter with a multinomial distribution.

The aim of the present work is to propose a how‐to guide for calculating the correct VC matrices of dependant estimates with multinomial distribution and how to use them when covariates are tested in post hoc analyses. We first briefly describe the general statistical requirements on link functions and the Delta method and refer to corresponding theoretical papers. Second, we focus on the specificities of CR analyses and how to assess the effect of covariates on parameters involved in the multinomial distribution (e.g., multinomial logit link function), a case in which covariates could not be integrated directly in a new model. We provide R code (Team, 2015) in the (Appendix S1) Supporting information to reproduce these post hoc analyses and serve as practical guide for other potential applications.

## 2 HOW DOES THE DELTA METHOD WORK IN SIMPLE CASES?

Such as other biological quantities, demographic probabilities (e.g., survival or detection probabilities) can, by definition, vary between 0 and 1. Commonly in statistical analyses, these quantities are transformed with link functions, which provides a mapping of (0,1) space to the real number axis, so between −∞ and +∞, which is useful for most basic statistics (e.g., linear model). In CR analyses, several link functions can be used (Table 1), but the most commonly used is the logit function, such as:

$$\beta =\log it\left(\theta \right)=\ln\left(\frac{\theta }{1-\theta }\right),$$

where ***θ*** can be estimates of any demographic parameters and *β*s represent the transformed ***θ*** (hereafter named mathematical parameters). To get back the demographic parameters ***θ***, that is, on the [0,1] probability scale, the back‐transformation, also called the inverse logit function (Table 1), is:

$$\theta =\text{inverse logit}\;\left(\beta \right)=\frac{e^{\beta }}{1+e^{\beta }}=\frac{1}{1+e^{-\beta }}=\text{logistic}\left(\beta \right),$$

**Table 1:** Rules for calculating derivatives of several link functions, and their inverse, used in CMR analyses

Link	Function	Derivative of function	Inverse function (back‐transformations)	Derivative of inverse function
Logit	$\beta =\ln\left(\frac{\theta }{1-\theta }\right)\text{logit}\left(\beta \right)$	$\frac{1}{\left(1-\theta \right)\theta }$	$\theta =\frac{e^{\beta }}{1+e^{\beta }}=\frac{1}{1+e^{-\beta }}$ $\text{inverse logit}\left(\beta \right)$ $\text{logistic}\left(\beta \right)$	$\frac{e^{-\beta }}{{\left(1+e^{-\beta }\right)}^{2}}=\left(1-\frac{1}{1+e^{-\beta }}\right)*\frac{1}{1+e^{-\beta }}=\left(1-\theta \right)\theta$
Multinomial logit (Example with *β*_*A*1_ or *θ*_*AB*_ of the prerequisite section)	$\beta_{A1}=\ln\left(\frac{\theta_{\mathrm{AB}}}{1-\theta_{\mathrm{AB}}-\theta_{\mathrm{AC}}-\theta_{\mathrm{AD}}-\theta_{\mathrm{AE}}}\right)$	$\frac{\partial \beta_{A1}}{\partial \theta_{\mathrm{AB}}}=\frac{1-\theta_{\mathrm{AC}}-\theta_{\mathrm{AD}}-\theta_{\mathrm{AE}}}{\left(1-\theta_{\mathrm{AB}}-\theta_{\mathrm{AC}}-\theta_{\mathrm{AD}}-\theta_{\mathrm{AE}}\right)\theta_{\mathrm{AB}}}\frac{\partial \beta_{A1}}{\partial \theta_{\mathrm{AC}}}=\frac{\partial \beta_{A1}}{\partial \theta_{\mathrm{AD}}}=\frac{\partial \beta_{A1}}{\partial \theta_{\mathrm{AE}}}=\frac{1}{1-\theta_{\mathrm{AB}}-\theta_{\mathrm{AC}}-\theta_{\mathrm{AD}}-\theta_{\mathrm{AE}}}$	$\theta_{\mathrm{AB}}=\frac{e^{\beta_{A1}}}{1+e^{\beta_{A1}}+e^{\beta_{A2}}+e^{\beta_{A3}}+e^{\beta_{A4}}}$	$\frac{\partial \theta_{\mathrm{AB}}}{\partial \beta_{A1}}=\left(1-\theta_{\mathrm{AB}}\right)\theta_{\mathrm{AB}}$ $\frac{\partial \theta_{\mathrm{AB}}}{\partial \beta_{A2}}=-\theta_{\mathrm{AB}}\theta_{\mathrm{AC}}$
Arcsin	$\beta =\arcsin\left(2\theta -1\right)$	$\frac{2}{\sqrt{1-{\left(2\theta -1\right)}^{2}}}$	$\theta =\frac{\sin\left(\beta \right)+1}{2}$	$\frac{\cos\left(\beta \right)}{2}$
Log: Natural Logarithm	$\beta =\ln\left(\theta \right)$	1/*θ*	*θ* = *e* ^*β*^	*e* ^*β*^
Log‐log	$\beta =\ln\left(-\ln(\theta )\right)$	$\frac{1}{\theta \ln\left(\theta \right)}$	$\theta =e^{-e^{\beta }}$	$-e^{\beta -e^{\beta }}$
Complementary log‐log	*β* = ln ( ‐ ln (1 ‐ *θ*))	$\frac{1}{\ln\left(1-\theta \right)\left(\theta -1\right)}$	$\theta =1-e^{-e^{\beta }}$	$e^{\beta -e^{\beta }}$
Identity	*β* = *θ*	1	*θ* = *β*	1

Notes

*θ* refers to any biological parameter (e.g., survival probability) and *β* represents the transformed *θ*, that is, a mathematical parameter. Some of these derivatives are needed in the approximation of the variance–covariance matrix using the Delta method. Logit, arcsin, log‐log, and complementary log‐log link functions constrain parameters to the interval [0, 1], whereas identity and log link functions do not.

Then, to assess the impact of a covariate ***x*** on demographic parameters, CR models are constructed to constrain estimates of these parameters to be a function of ***x***. In the case of a linear relation, the logit link function, as well as the other link functions of Table 1, can thus be written as:

$$\text{logit}\left(\theta \right)=\ln\left(\frac{\theta }{1-\theta }\right)=\beta_{i}+\beta_{s}*x,$$

where ***β***_*i*_ is the intercept and ***β***_*s*_ the slope of this linear relation.

Similarly, the back‐transformation is:

$$\theta =\text{inverse logit}\left(\beta_{i}+\beta_{s}*x\right)=\frac{e^{\left(\beta_{i}+\beta_{s}*x\right)}}{1+e^{\left(\beta_{i}+\beta_{s}*x\right)}}=\frac{1}{1+e^{-\left(\beta_{i}+\beta_{s}*x\right)}}=\text{logistic}\left(\beta_{i}+\beta_{s}*x\right),$$

Several of these biological quantities cannot be measured simply but rather need to be estimated from models. Estimates of these quantities ***θ*** derived from models, as well as the mathematical parameters (β), come with variance and covariance. This non‐independence is expressed through a VC matrix (*V*(***θ***) and V(β) respectively), which needs to be taken into account to perform proper analyses. However, as stated above, the ***θ*** are very often transformed to be used in statistical analyses, for example, with link functions in the case of transformations of probability. These scale modifications, when using a link function, influence the shape of the likelihood and thus the estimate of the variance (Cooch & White, 2016). It is not as simple as back transforming the V(β) to obtain *V*(***θ***).

Yet, a convenient and straightforward method for obtaining variance and covariance of one or more transformed demographic parameters is the Delta method that uses the one‐dimensional Taylor series approximation (Cooch & White, 2016; Williams et al., 2002). We can approximate *V*(***θ***), the VC matrix of any demographic parameters, by:

$$V\left(\theta \right)=D_{\theta }*V\left(\beta \right)*{D_{\theta }}^{T},$$

where ***β*** represent the mathematical parameters, that is, the transformed ***θ*** after applying the link function, *V*(***β***) is the VC matrix of the *β*s, *D*_***θ***_ is the matrix of the first derivatives of the demographic parameters (***θ***) and *D*_***θ***_^*T*^ is the transpose of *D*_***θ***_.

The logit link function (such as others, see Table 1) is well adapted to variables with binomial distributions, thus convenient in CR analyses where several phenomena are essentially binary (e.g., survived or died, breeder versus non‐breeder, captured or not). However, other phenomena, namely transitions in multistate models, are essentially multinomial.

## 3 HOW TO ASSESS THE EFFECT OF COVARIATES ON PARAMETERS WITH MULTINOMIAL DISTRIBUTION

**Figure 2:**
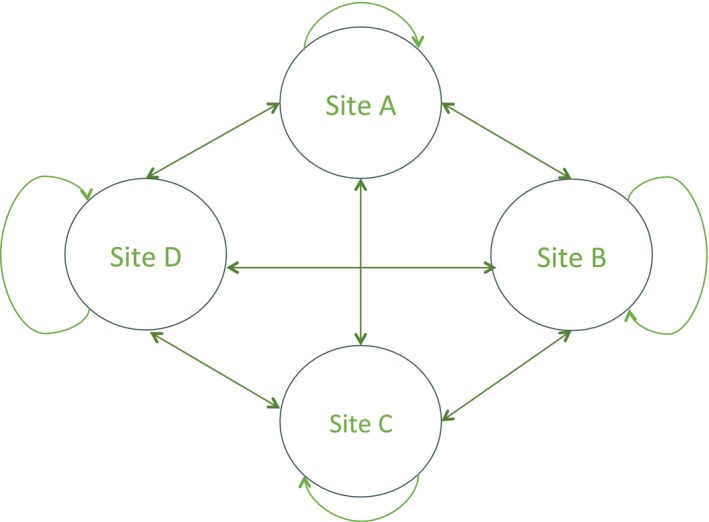
Schematic of the possible transitions between different breeding locations

In more complex CR models, such as multistate (Nichols & Kendall, 1995) or multi‐event (Pradel, 2005) models, the probabilities of interest can have more than two modalities. As an example, we could study the impact of the distance separating any two locations on the frequency of movements between them. In a situation where there are 4 sites, starting from any one site, we have four potential movements including the probability of not moving (Figure 2). Since the associated probabilities must sum to 1, the logit link function is no longer satisfactory and has to be replaced by the multinomial (or generalized) logit (McFadden, 1968) link function. The implementation of this constraint, that the sum of the probabilities of movement from a given departure site is equal to 1, forces in effect a parameterization, where there is no direct correspondence between an actual parameter (***β***) and a particular movement (***θ***).

$$\begin{array}{cc} \theta_{\mathrm{AB}} & =\frac{e^{\beta_{A1}}}{1+e^{\beta_{A1}}+e^{\beta_{A2}}+e^{\beta_{A3}}};\;\theta_{\mathrm{AC}}=\frac{e^{\beta_{A2}}}{1+e^{\beta_{A1}}+e^{\beta_{A2}}+e^{\beta_{A3}}};\\ \theta_{\mathrm{AD}} & =\frac{e^{\beta_{A3}}}{1+e^{\beta_{A1}}+e^{\beta_{A2}}+e^{\beta_{A3}}};\;\theta_{\mathrm{AA}}=1-\theta_{\mathrm{AB}}-\theta_{\mathrm{AC}}-\theta_{\mathrm{AD}}, \end{array}$$

Although there are as many mathematical parameters (3***β***) as independent demographic parameters (3 ***θ***), there is no one‐to‐one correspondence as can be seen from the equations above (any individual *θ* is a function of all the components of ***β*** through the denominator).

An alternative parameterization separates the probability to disperse from the probabilities of settlement conditional on dispersal (Grosbois & Tavecchia, 2003). If we take the probability of moving to site D as the complement, then we have:

$$\beta_{A1}^{\prime }=\ln\left(\frac{\theta_{\mathrm{AB}}^{\prime }}{1-\theta_{\mathrm{AB}}^{\prime }-\theta_{\mathrm{AC}}^{\prime }}\right)\;\text{and}\;\beta_{A2}^{\prime }=\ln\left(\frac{\theta_{\mathrm{AC}}^{\prime }}{1-\theta_{\mathrm{AB}}^{\prime }-\theta_{\mathrm{AC}}^{\prime }}\right),$$

where $\theta_{\mathrm{AB}}^{\prime }$ is the transition from A to B, that is, the probability that an individual leaving site A settles on site B the subsequent year. Inverting the multinomial logit function, we obtain the probabilities of settlement as:

$$\theta_{\mathrm{AB}}^{\prime }=\frac{e^{\beta_{A1}^{\prime }}}{1+e^{\beta_{A1}^{\prime }}+e^{\beta_{A2}^{\prime }}};\;\theta_{\mathrm{AC}}^{\prime }=\frac{e^{\beta_{A2}^{\prime }}}{1+e^{\beta_{A1}^{\prime }}+e^{\beta_{A2}^{\prime }}};\;\theta_{\mathrm{AD}}^{\prime }=1-\theta_{\mathrm{AB}}^{\prime }-\theta_{\mathrm{AC}}^{\prime },$$

There is still no one‐to‐one correspondence between the individual *β*′ and the individual *θ*′.

**Figure 3:**
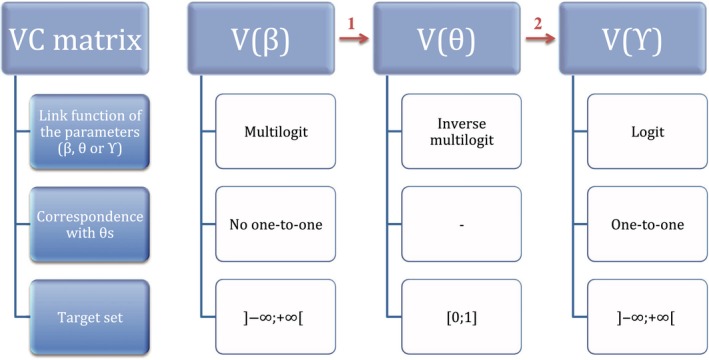
General approach for assessing the impact of a covariate on parameters with a multinomial distribution. “VC” means variance–covariance, “*β*” are the mathematical parameters, “*θ*” the estimates of biological quantities and “ϒ” the one‐to‐one individual transformed estimates. Red arrows indicate the steps (1 and 2) where the Delta method is applied

This absence of a one‐to‐one correspondence between demographic and mathematical parameters renders obscure, if at all possible, the way to implement a desired constraint on demographic parameters. Therefore, we propose to extract estimates of the demographic parameters from the *unconstrained* model along with their VC matrix, and then run post hoc analyses. As stated above, because the estimates will not be independent, traditional statistical techniques are not appropriate. We propose to use instead the generalized least square (GLS; Aitken 1936) framework. However, for this method to perform properly we need to ensure that all values in the set of real numbers are admissible. This is ensured if we work on the logit scale. The first step is thus to calculate the simple logit of ***θ′*** (or ***θ*** **)** above. In this transformation, each element of ***θ′*** is mapped one‐to‐one onto an element of a new vector (thereafter called ***γ***), which corresponds to a transition and only one and which takes its value between −∞ and +∞. Then, we calculate the associated VC matrix (V(***γ***)), for example, with the Delta method, which will be used in the GLS analyses. Indeed, the mathematical parameters (***β′***) cannot be used because they do not correspond to any particular transition, and the transitions (***θ′***) themselves have the disadvantage to vary between 0 and 1, on which regression cannot be made directly (Figure 3).

Program MARK (White & Burnham, 1999) provides the VC matrix for the demographic parameters (V(***θ***′)). We only need to derive the VC matrix V(***γ***) of the logits of ***θ*** (***γ***), prior to the GLS analysis (Step 2, Figure 3). If instead program E‐SURGE (Choquet, Rouan, & Pradel, 2009) is used, only the VC matrix of the mathematical parameters (V(***β***′)) is directly available, so that there is an additional step in this case (Step 1, Figure 3). We describe the steps required when only the VC matrix of the mathematical parameters is provided as part of step 1 in the analyses.

Step 1: As per the Delta method, we start from $V\left(\theta^{\prime }\right)=D_{\theta }^{\prime }*V\left(\beta^{\prime }\right)*{D_{\theta }^{\prime }}^{T}$. Using the same example as above: $V\left(\beta^{\prime }\right)=V\left(\begin{array}{c} \beta_{A1}^{\prime }\\ \beta_{A2}^{\prime } \end{array}\right)$ and $V\left(\theta^{\prime }\right)=V\left(\begin{array}{c} \theta_{\mathrm{AB}}^{\prime }\\ \theta_{\mathrm{AC}}^{\prime } \end{array}\right)$. In order to calculate *V*(***θ′***), we first need to calculate the matrix of the first derivatives of ***θ′*** with respect to ***β′*** ($D_{\theta }=\frac{{\delta \theta }^{\prime }}{{\delta \beta }^{\prime }}$). Depending on which ***β′*** we derived with respect to, the first derivative of ***θ′*** will differ. After some algebra based on Table 1, we can write the partial first derivative of ***θ***_*AB*_^*′*^ with respect to each ***β′*** such as:

$$\frac{\partial \theta_{\mathrm{AB}}^{\prime }}{\partial \beta_{A1}^{\prime }}=\left(1-\theta_{\mathrm{AB}}^{\prime }\right)*\theta_{\mathrm{AB}}^{\prime },\frac{\partial \theta_{\mathrm{AB}}^{\prime }}{\partial \beta_{A2}^{\prime }}=-\theta_{\mathrm{AB}}^{\prime }*\theta_{\mathrm{AC}}^{\prime }$$

and any other $\frac{\partial \theta_{\mathrm{AB}}^{\prime }}{\partial \beta_{\mathrm{ij}}^{\prime }}=0$ with *i* ≠ *A* and j from 1 to 2. This was applied in all cases to get $D_{\theta }^{\prime }$ (Table 2), then to take the transpose, ${D_{\theta^{\prime }}}^{T}$, and to consequently calculate $V\left(\theta^{\prime }\right)$.

**Table 2:** Matrix ($D_{\theta_{X}}$) of the first derivatives of the biological parameters (*θ*s) (in rows) with respect to the mathematical parameters (*β*s) (in columns)

	*β*_*A*1_	*β*_*A*2_	*β*_*B*1_	*β*_*B*2_	*β*_*C*1_	*β*_*C*2_
*θ*_*AB*_	(1 − *θ*_*AB*_)*θ*_*AB*_	−*θ*_*AB*_ *θ*_*AC*_	0	0	0	0
*θ*_*AC*_	−*θ*_*AC*_ *θ*_*AB*_	(1 − *θ*_*AC*_)*θ*_*AC*_	0	0	0	0
*θ*_*BA*_	0	0	(1 − *θ*_*BA*_)*θ*_*BA*_	−*θ*_*BA*_ *θ*_*BC*_	0	0
*θ*_*BC*_	0	0	−*θ*_*BC*_ *θ*_*BA*_	(1 − *θ*_*BC*_)*θ*_*BC*_	0	0
*θ*_*CA*_	0	0	0	0	(1 − *θ*_*CA*_)*θ*_*CA*_	−*θ*_*CA*_ *θ*_*CB*_
*θ*_*CB*_	0	0	0	0	−*θ*_*CB*_ *θ*_*CA*_	(1 − *θ*_*CB*_)*θ*_*CB*_

For example, *θ*_*AB*_ is the transition from A to B, that is, the probability that an individual leaving site A settles on site B the subsequent year.

Step 2: In our example, V(***γ***) = $V\left(\begin{array}{c} \gamma_{\mathrm{AB}}\\ \gamma_{\mathrm{AC}}\\ \gamma_{\mathrm{AD}} \end{array}\right)$. To calculate ***γ*** and V(***γ***), an additional transformation on the logit scale of ***θ′*** and their corresponding VC matrix had to be calculated. Thus, $\gamma =logit\;\left(\theta^{\prime }\right)=\ln\left(\frac{\theta^{\prime }}{{1-\theta }^{\prime }}\right)$. Then, using $V\left(\theta^{\prime }\right)$ either previously calculated or directly provided by the program MARK, the Delta method gives for example:

$$\begin{array}{cc} & \text{cov}\left(\gamma_{\mathrm{AB}},\gamma_{\mathrm{AC}}\right)=\text{cov}\left(\text{logit}\left(\theta_{\mathrm{AB}}^{\prime }\right),\;\text{logit}\left(\theta_{\mathrm{AC}}^{\prime }\right)\right)=\frac{\text{cov}\left(\theta_{\mathrm{AB}}^{\prime },\theta_{\mathrm{AC}}^{\prime }\right)}{\theta_{\mathrm{AB}}^{\prime }\left(1-\theta_{\mathrm{AB}}^{\prime }\right)\theta_{\mathrm{AC}}^{\prime }\left(1-\theta_{\mathrm{AC}}^{\prime }\right)}\\ & \;\text{and}\;\;\text{var}\left(\gamma_{\mathrm{AB}}\right)=\text{var}\left(\text{logit}\left(\theta_{\mathrm{AB}}^{\prime }\right)\right)=\frac{\text{var}\left(\theta_{\mathrm{AB}}^{\prime }\right)}{{\left(\theta_{\mathrm{AB}}^{\prime }\right)}^{2}{\left(1-\theta_{\mathrm{AB}}^{\prime }\right)}^{2}} \end{array}$$

After applying these formula to all cases, we get V(***γ***), presented in Table 3. The one‐to‐one individual transformed estimates (***γ***) and their VC matrix (V(***γ***)) can now be used to perform a GLS linear regression between the movement probability estimates and a covariate (e.g., distance between sites *A*,* B*,* C*, and *D*). This procedure is implemented in MATLAB with the function lscov and in R with the function lm.gls of the library MASS. The R script that allows doing these calculations is detailed in the supporting information (Appendix S1).

**Table 3:** Variance–covariance matrix U(*γ*_*X*_) of the individual mathematical parameters (*γ*_*X*_)

	*γ*_*Y*1 → *Y*2_	…	*γ*_*Y*2 → *Y*3_	…	*γ*_*Y*4 → *Y*5_
*γ*_*Y*1 → *Y*2_	$\frac{\text{var}\left(\theta_{Y1\to Y2}\right)}{{\left(\theta_{Y1\to Y2}\right)}^{2}{\left(1-\theta_{Y1\to Y2}\right)}^{2}}$	…	$\frac{\text{cov}\left(\theta_{Y1\to Y2},\theta_{Y2\to Y3}\right)}{\theta_{Y1\to Y2}\left(1-\theta_{Y1\to Y2}\right)\theta_{Y2\to Y3}\left(1-\theta_{Y2\to Y3}\right)}$	…	$\frac{\text{cov}\left(\theta_{Y1\to Y2},\theta_{Y4\to Y5}\right)}{\theta_{Y1\to Y2}\left(1-\theta_{Y1\to Y2}\right)\theta_{Y4\to Y5}\left(1-\theta_{Y4\to Y5}\right)}$
…	…	…	…	…	…
*γ*_*Y*2 → *Y*3_	$\frac{\mathrm{cov}\left(\theta_{Y2\to Y3},\theta_{Y1\to Y2}\right)}{\theta_{Y2\to Y3}\left(1-\theta_{Y2\to Y3}\right)\theta_{Y1\to Y2}\left(1-\theta_{Y1\to Y2}\right)}$	…	$\frac{\text{var}\left(\theta_{Y2\to Y3}\right)}{{\left(\theta_{Y2\to Y3}\right)}^{2}{\left(1-\theta_{Y2\to Y3}\right)}^{2}}$	…	$\frac{\text{cov}\left(\theta_{Y2\to Y3},\theta_{Y4\to Y5}\right)}{\theta_{Y2\to Y3}\left(1-\theta_{Y2\to Y3}\right)\theta_{Y4\to Y5}\left(1-\theta_{Y4\to Y5}\right)}$
…	…	…	…	…	…
*γ*_*Y*4 → *Y*5_	$\frac{\mathrm{cov}\left(\theta_{Y4\to Y5},\theta_{Y1\to Y2}\right)}{\theta_{Y4\to Y5}\left(1-\theta_{Y4\to Y5}\right)\theta_{Y1\to Y2}\left(1-\theta_{Y1\to Y2}\right)}$	…	$\frac{\text{cov}\left(\theta_{Y4\to Y5},\theta_{Y2\to Y3}\right)}{\theta_{Y4\to Y5}\left(1-\theta_{Y4\to Y5}\right)\theta_{Y2\to Y3}\left(1-\theta_{Y2\to Y3}\right)}$	…	$\frac{\text{var}\left(\theta_{Y4\to Y5}\right)}{{\left(\theta_{Y4\to Y5}\right)}^{2}{\left(1-\theta_{Y4\to Y5}\right)}^{2}}$

By way of illustration, we reproduce here the example of the role of distance on conditional settlement probabilities (Fernández‐Chacón et al., 2013), that is, decision on where to go once animals have left their previous site. A long‐lived seabird was monitored in four sites. In this study, settlement probabilities were estimated with multi‐event mark–recapture models in E‐SURGE, taking into account as the complement the settlement on a catchall fifth site (ghost location), which serves for all individuals that go to unmonitored sites or skip breeding altogether. Following the approach presented above with five sites instead of four, we started from the ***β*** and V(***β***) to get the ***θ*** and V(***θ***) (Step 1), then to obtain the ***γ*** and V(***γ***) (Step 2), to finally test, with a GLS linear regression, the link between distance separating each of the sites and settlement probabilities. In this numerical example, distance to the destination site did not influence settlement choices (Fernández‐Chacón et al., 2013). R routines, numerical steps, variance–covariance matrices, and results are provided in supporting information (Appendix S1).

## 4 CONCLUSION

Although integrating the covariate directly into a given model is the preferred method, this is not always possible. The two‐step approach presented in this paper works well in the context of CR analyses and has the additional advantage of allowing manipulating the biological quantities themselves rather than the compounded parameters of multinomial logits. Additionally, our method is impervious to the difficulties that may arise in a direct analysis when the same covariate is hypothesized to act on quantities derived from more than one multinomial. For example, when testing the effect of distance on movement probability, the probability to move from site A to site B should be equal to that to move from B to A if distance is the sole determinant. Although the two quantities belong to two different multinomials, this is not an obstacle in our approach.

One may wonder how good is this alternative of post hoc analysis. Preliminary analyses without multinomial distribution (not presented) showed that integrating the covariate into the models or running a posteriori analyses accounting for correlated estimates provided the same biological conclusions. For example, assessing the effect of the winter NAO on annual survival either comparing models with the integrated covariate (Guéry et al., 2017) or with the GLS technique performing a linear regression between the winter NAO and annual survival led to similar conclusions. However, the interpretation of the details needs to be careful with the extreme case of parameters estimated at the boundaries (0 or 1), where the logit transformation is not linear anymore for these limits (logit(0) = −∞ and logit(1) = +∞). Indeed, the Delta method assumes that the transformation of variables is approximately linear over the expected range of the parameter; otherwise, it could fail to correctly approximate the variance (Cooch & White, 2016; Williams et al., 2002).

Our study mostly uses examples from a CR framework but what we propose goes beyond CR models and can be used in any framework as long as one deals with parameter estimates that come from the same model. We hope that this paper will serve as reference and guideline in further needed investigations, not only in population biology but also in all analyses dealing with correlated estimates and their variances and covariances.

## CONFLICT OF INTEREST

None declared.

## AUTHORS’ CONTRIBUTION

Loreleï Guéry, Lauriane Rouan and Roger Pradel set up the idea. Loreleï Guéry, Lauriane Rouan, Roger Pradel, Joël Bêty and Sébastien Descamps designed methodology. Loreleï Guéry conducted analyses and wrote the paper. Albert Fernández‐Chacón and Grant Gilchrist provided the data. All authors contributed critically to the drafts and gave final approval for publication.

## Data Availability

Data used are provided in the supporting information (Appendix S1).
