# Supplementary material for: Covariate and multinomial: Accounting for distance in movement in capture–recapture analyses
Source: Ecol Evol. 2019 Feb 5;9(2):818–24. doi: 10.1002/ece3.4827 (PMC6362441; doi:10.1002/ece3.4827)
Supplement: Supplementary file 1 [file ECE3-9-818-s001.docx]

**Supporting Information**

R routine, numerical application and results of post-hoc analyses when the covariate cannot be integrated in the model (multinomial issue) : example of the probabilities of settlement conditional on dispersal

**# Initial values**

nsites = 5

chat = 1 #chat is the coeficient of overdispersion, 1 by default

**###########################################################################**

**# Beta and variance-covariance matrix of the beta**

**###########################################################################**

**# Import beta provided by the software (output of E-SURGE program for example)**

beta <-c(0.4321434,0.2036048,0.1832377,0.462086,0.1772177,0.1834787,0.277495,0.2440377,0.2392337,0.4753669,0.1748777,0.1748777)

**# Import V(beta) provided by the software (output of E-SURGE program for example)**

nbeta = (nsites-1-1)*(nsites-1) **# Number of mathematical parameters (beta) if we take the probability of moving to site D as the complement**

V_beta <- matrix(0, nbeta, nbeta)

V_beta[1,]<-c(0.20614054,0.15905685,0.03321758,1.04026856,-0.05664936,1.14629378,-0.01732456,-0.01418431,-0.03188666,0,0,0)

V_beta[2,]<-c(0.15905685,0.17940281,0.05448564,1.01921411,-0.05570602,1.15025645,-0.01077763,-0.02180492,-0.03172129,0,0,0)

V_beta[3,]<-c(0.03321758,0.05448564,0.15419649,0.67031994,-0.03361497,0.69258843,-0.00492457,-0.01481698,-0.01980359,0,0,0)

V_beta[4,]<-c(1.04026856,1.01921411,0.67031994,7.30974251,-0.38204743,7.87568008,-0.08578104,-0.12440104,-0.22089041,0,0,0)

V_beta[5,]<-c(-0.05664936,-0.05570602,-0.03361497,-0.38204743,0.02101213,- 0.41914279,0.00456552,0.00640252,0.01176308,0,0,0)

V_beta[6,]<-c(1.14629378,1.15025645,0.69258843,7.87568008,-0.41914279,8.72916172,-0.09225856,-0.14225517,-0.24183528,0,0,0)

V_beta[7,]<-c(-0.01732456,-0.01077763,-0.00492457,-0.08578104,0.00456552,- 0.09225856,0.03589406,0.02909819,0.0022974,0,0,0)

V_beta[8,]<-c(-0.01418431,-0.02180492,-0.01481698,-0.12440104,0.00640252,-.14225517,0.02909819,0.42137069,0.00252894,0,0,0)

V_beta[9,]<-c(-0.03188666,-0.03172129,-0.01980359,-0.22089041,0.01176308,-0.24183528,0.0022974,0.00252894,0.00677493,0,0,0)

V_beta[10,]<-c(0,0,0,0,0,0,0,0,0,0,0,0)

V_beta[11,]<-c(0,0,0,0,0,0,0,0,0,0,0,0)

V_beta[12,]<-c(0,0,0,0,0,0,0,0,0,0,0,0)

Vhat <- chat* V_beta

**###########################################################################**

**# Theta and variance-covariance matrix of the theta**

**###########################################################################**

**# Import theta provided by the software (output of E-SURGE program for example)**

**# They could also be calculated from the beta**

theta <- c(0.870182516,0.117605721,0.012209383,0.958372186,0.000000005,0.034719883,0.148362582,0.019882038,0.000000329,1,0,0)

ntheta <- as.numeric(length(theta))

**# Step 1: calcul of V(theta) from V(beta) if not provided by the software (E-SURGE program for example)**

D_theta <- diag(x = 1, nrow = ntheta, ncol = nbeta)

for (i in 1:(nsites-3)){

for (j in 2:(nsites-2)){

for (k in 0:(nsites-2)){

D_theta[i+k*(nsites-2),j+k*(nsites-2)] <- D_theta[j+k*(nsites-2),i+k*(nsites-2)] <- -theta[i+k*(nsites-2)]*theta[j+k*(nsites-2)]

}

}

}

diag(D_theta) <- theta*(1-theta)

V_theta <- D_theta %*% Vhat %*% t(D_theta) # Delta method

**###########################################################################**

**# Gamma and variance-covariance matrix of the gamma**

**###########################################################################**

**# Calcul of the gamma (one-to-one), called logit_theta for R language issues**

logit_theta <- log(theta /(1- theta))

**# Step 2: Calculate V(gammas) from V(thetas)**

V_gamma <- matrix(0, nrow = length(logit_theta), ncol = length(logit_theta))

for (i in 1:length(logit_theta)){

for (j in 1:length(logit_theta)){

V_gamma[i,j] <- V_theta[i,j]/(logit_theta[i]*(1-logit_theta[i])*logit_theta[j]*(1-logit_theta[j]))

}

}

diag(V_gamma) <- diag(V_theta)/(theta^2*(1-theta)^2)

**###########################################################################**

**# Generalized Least Square (GLS) linear regression**

**###########################################################################**

**# Covariate, e.g. standardized distance (distN)**

distN <- c(-1.3491,1.0343,-0.6367,-1.3491,0.7592,-0.8010,1.0343,0.7592,0.9933,-0.6367,-0.8010,0.9933)

**#In case of probabilities estimated at 0 or 1, asymptotic variances could not be computed, leading to a non-invertible variance matrix as well as infinite values of the logit_theta**

**# To solve this problem a solution is to add a small quantity on the diagonal (0.000000001 here).**

V_gamma[which(is.na(V_gamma))] <- 0

diag(V_gamma) <- diag(V_gamma) + 0.000000000001

theta_aug <- ifelse(theta == 0,0.000000000001,theta)

theta_aug <- ifelse(theta_aug == 1,(1-0.000000000001),theta_aug)

logit_theta <- log(theta_aug /(1- theta_aug))

logit_theta

**# GLS analyses**

library(MASS)

data <- as.data.frame(cbind(X=logit_theta,Y=distN))

model <- lm.gls(X~Y, data = data, as.matrix(V_gamma), inverse = TRUE)

summary(model)

u<-coef(model)

u

ntheta = 12

D_theta

| 0.11296490 | -0.102338442 | -0.010624392 | 0 | 0 | 0 | 0 | 0 | 0 | 0 | 0 | 0 |
| --- | --- | --- | --- | --- | --- | --- | --- | --- | --- | --- | --- |
| -0.10233844 | 0.103774615 | -0.001435893 | 0 | 0 | 0 | 0 | 0 | 0 | 0 | 0 | 0 |
| -0.01062439 | -0.001435893 | 0.012060314 | 0 | 0 | 0 | 0 | 0 | 0 | 0 | 0 | 0 |
| 0 | 0 | 0 | 3.989494e-02 | -4.791861e-09 | -3.327457e-02 | 0 | 0 | 0 | 0 | 0 | 0 |
| 0 | 0 | 0 | -4.791861e-09 | 5.000000e-09 | -1.735994e-10 | 0 | 0 | 0 | 0 | 0 | 0 |
| 0 | 0 | 0 | -3.327457e-02 | -1.735994e-10 | 3.351441e-02 | 0 | 0 | 0 | 0 | 0 | 0 |
| 0 | 0 | 0 | 0 | 0 | 0 | 1.263511e-01 | -2.949750e-03 | -4.881129e-08 | 0 | 0 | 0 |
| 0 | 0 | 0 | 0 | 0 | 0 | -2.949750e-03 | 1.948674e-02 | -6.541191e-09 | 0 | 0 | 0 |
| 0 | 0 | 0 | 0 | 0 | 0 | -4.881129e-08 | -6.541191e-09 | 3.289999e-07 | 0 | 0 | 0 |
| 0 | 0 | 0 | 0 | 0 | 0 | 0 | 0 | 0 | 0 | 0 | 0 |
| 0 | 0 | 0 | 0 | 0 | 0 | 0 | 0 | 0 | 0 | 0 | 0 |
| 0 | 0 | 0 | 0 | 0 | 0 | 0 | 0 | 0 | 0 | 0 | 0 |

V_theta

| 8.880393e-04 | -7.769525e-04 | -1.110734e-04 | 9.587309e-05 | -3.164330e-11 | -5.451340e-05 | -1.036261e-04 | 1.769281e-05 | -1.383425e-11 | 0 | 0 | 0 |
| --- | --- | --- | --- | --- | --- | --- | --- | --- | --- | --- | --- |
| -7.769525e-04 | 7.063947e-04 | 7.054893e-05 | -1.013345e-04 | 8.062437e-12 | 9.064324e-05 | 8.592337e-05 | -1.734454e-05 | -2.719134e-11 | 0 | 0 | 0 |
| -1.110734e-04 | 7.054893e-05 | 4.051994e-05 | 5.469176e-06 | 2.356793e-11 | -3.612064e-05 | 1.769790e-05 | -3.488627e-07 | 4.100289e-11 | 0 | 0 | 0 |
| 9.587309e-05 | -1.013345e-04 | 5.469176e-06 | 3.894080e-04 | -1.522525e-10 | -1.880036e-04 | -4.384479e-05 | -3.432662e-06 | -2.331306e-10 | 0 | 0 | 0 |
| -3.164330e-11 | 8.062437e-12 | 2.356793e-11 | -1.522525e-10 | 2.007720e-16 | -1.112564e-10 | 5.491896e-11 | 1.139426e-11 | 3.551709e-16 | 0 | 0 | 0 |
| -5.451340e-05 | 9.064324e-05 | -3.612064e-05 | -1.880036e-04 | -1.112564e-10 | 3.324928e-04 | -2.817605e-05 | -1.154066e-05 | -2.326633e-10 | 0 | 0 | 0 |
| -1.036261e-04 | 8.592337e-05 | 1.769790e-05 | -4.384479e-05 | 5.491896e-11 | -2.817605e-05 | 5.550109e-04 | 3.429923e-05 | -1.400536e-10 | 0 | 0 | 0 |
| 1.769281e-05 | -1.734454e-05 | -3.488627e-07 | -3.432662e-06 | 1.139426e-11 | -1.154066e-05 | 3.429923e-05 | 1.569755e-04 | -6.167473e-11 | 0 | 0 | 0 |
| -1.383425e-11 | -2.719134e-11 | 4.100289e-11 | -2.331306e-10 | 3.551709e-16 | -2.326633e-10 | -1.400536e-10 | -6.167473e-11 | 7.707820e-16 | 0 | 0 | 0 |
| 0 | 0 | 0 | 0 | 0 | 0 | 0 | 0 | 0 | 0 | 0 | 0 |
| 0 | 0 | 0 | 0 | 0 | 0 | 0 | 0 | 0 | 0 | 0 | 0 |
| 0 | 0 | 0 | 0 | 0 | 0 | 0 | 0 | 0 | 0 | 0 | 0 |

V_gamma

| 6.958972e-02 | -7.445601e-05 | -2.729899e-06 | 8.331738e-06 | -4.793087e-14 | -2.207380e-06 | -1.256868e-05 | 5.396859e-07 | -3.388545e-14 | 0 | 0 | 0 |
| --- | --- | --- | --- | --- | --- | --- | --- | --- | --- | --- | --- |
| -7.445601e-05 | 6.559416e-02 | 4.899823e-07 | -2.488569e-06 | 3.451066e-15 | 1.037200e-06 | 2.944998e-06 | -1.495065e-07 | -1.882096e-14 | 0 | 0 | 0 |
| -2.729899e-06 | 4.899823e-07 | 2.785811e-01 | 3.444652e-08 | 2.587258e-15 | -1.060020e-07 | 1.555704e-07 | -7.712291e-10 | 7.278751e-15 | 0 | 0 | 0 |
| 8.331738e-06 | -2.488569e-06 | 3.444652e-08 | 2.446636e-01 | -5.909966e-14 | -1.950863e-06 | -1.362779e-06 | -2.683256e-08 | -1.463336e-13 | 0 | 0 | 0 |
| -4.793087e-14 | 3.451066e-15 | 2.587258e-15 | -5.909966e-14 | 8.030879e+00 | -2.012243e-14 | 2.975256e-14 | 1.552430e-15 | 3.885767e-21 | 0 | 0 | 0 |
| -2.207380e-06 | 1.037200e-06 | -1.060020e-07 | -1.950863e-06 | -2.012243e-14 | 2.960186e-01 | -4.080584e-07 | -4.203360e-08 | -6.804675e-14 | 0 | 0 | 0 |
| -1.256868e-05 | 2.944998e-06 | 1.555704e-07 | -1.362779e-06 | 2.975256e-14 | -4.080584e-07 | 3.476508e-02 | 3.741941e-07 | -1.226932e-13 | 0 | 0 | 0 |
| 5.396859e-07 | -1.495065e-07 | -7.712291e-10 | -2.683256e-08 | 1.552430e-15 | -4.203360e-08 | 3.741941e-07 | 4.133838e-01 | -1.358805e-14 | 0 | 0 | 0 |
| -3.388545e-14 | -1.882096e-14 | 7.278751e-15 | -1.463336e-13 | 3.885767e-21 | -6.804675e-14 | -1.226932e-13 | -1.358805e-14 | 7.120984e-03 | 0 | 0 | 0 |
| 0 | 0 | 0 | 0 | 0 | 0 | 0 | 0 | 0 | 1,00E-12 | 0 | 0 |
| 0 | 0 | 0 | 0 | 0 | 0 | 0 | 0 | 0 | 0 | 1,00E-12 | 0 |
| 0 | 0 | 0 | 0 | 0 | 0 | 0 | 0 | 0 | 0 | 0 | 1,00E-12 |

logit_theta = 1.902573 -2.015301 -4.393266 3.136468 -19.113828 -3.325106 -1.747502 -3.897856 -14.927208 27.631043 -27.631021 -27.631021

> summary(model)

Length Class Mode

coefficients 2 -none- numeric

residuals 12 -none- numeric

effects 12 -none- numeric

rank 1 -none- numeric

fitted.values 12 -none- numeric

assign 0 -none- NULL

qr 5 qr list

df.residual 1 -none- numeric

terms 3 terms call

call 5 -none- call

xlevels 0 -none- list

> u<-coef(model)

> u

(Intercept) Y

-11.24279 -13.72049

In this numerical example, distance to the destination site did not influence settlement choices (Fernandez-Chacon *et al*. 2013).
